# Risk Factors for Mortality of COVID-19 Patient Based on Clinical Course: A Single Center Retrospective Case-Control Study

**DOI:** 10.3389/fimmu.2021.581469

**Published:** 2021-02-16

**Authors:** Jing Zhang, Zhihua Wang, Xiong Wang, Zhiquan Hu, Chunguang Yang, Ping Lei

**Affiliations:** ^1^Department of Oncology, Tongji Hospital, Tongji Medical College, Huazhong University of Science and Technology, Wuhan, China; ^2^Department of Urology, Tongji Hospital, Tongji Medical College, Huazhong University of Science and Technology, Wuhan, China; ^3^Department of Clinical Laboratory, Tongji Hospital, Tongji Medical College, Huazhong University of Science and Technology, Wuhan, China; ^4^Department of Immunology, Tongji Medical College, Huazhong University of Science and Technology, Wuhan, China

**Keywords:** COVID-19, SARS-CoV-2, prognosis, lymphocyte subsets, complement component 3, inflammatory factors, immune function

## Abstract

**Background:** Epidemiological factors, clinical characteristics, and risk factors for the mortality of COVID-19 patients have been studied, but the role of complementary systems, possible inflammatory and immune response mechanisms, and detailed clinical courses are uncertain and require further study.

**Methods:** In this single center, retrospective case-control study, we included all COVID-19 inpatients transferred or admitted to Wuhan Tongji Hospital from January 3 to March 30 2020 who had definite clinical outcomes (cured or deceased) with complete laboratory and radiological results. Clinical data were extracted from the electronic medical records, and compared between the cured and deceased patients. ROC curves were used to evaluate the prognostic value of the clinical parameters, and multivariable logistic regression analysis was performed to explore the risk factors for mortality. The correlation between the variables was evaluated by Spearman correlation analysis.

**Results:** 208 patients were included in this study, 182 patients were cured and discharged, 26 patients died from COVID-2019. Most patients had comorbidities, with hypertension as the most common chronic disease (80; 38%). The most common symptoms at onset were fever (149; 72%), cough (137; 66%), and dyspnea (113; 54%). Elevated leucocytes, neutrophils, inflammatory biomarkers (CRP, ferritin, IL6, IL8, procalcitonin), PT, D-dimer, myocardial enzymes, BUN, decreased lymphocyte and subsets (T cells, CD4 T cells, CD8 T cells, NK cells, T cells + B cells + NK cells), and immunological factors (C3, C4) indicated poor outcome. PT, C3, and T cells were confirmed as independent prognostic factors for mortality by logistic regression models. IL6 and CPR were positively correlated with neutrophils, but negatively with lymphocytes and lymphocyte subsets except B cells. IL8 and ferritin were negatively related to T cells and CD4 T cells. Positive associations existed between C3 and T cells, CD4 T cells, and CD8 T cells, whereas there was no significant correlation between C4 and lymphocyte subsets. PT was found positively correlated with IL6, IL8, and CRP. Reverse correlations were explored between C3, C4, and PT, CK-MB, total bilirubin.

**Conclusions:** T cells, C3, and PT were identified as independent prognostic factors for mortality. Decreased C3 and C4, dysregulation of lymphocyte subsets and cytokines may lead to death after SARS-CoV-2 infection.

## Introduction

The worldwide outbreak of coronavirus disease 2019 (COVID-19), with the first case confirmed in Wuhan, China, in December 2019, is caused by a new strain of coronavirus, Severe acute respiratory syndrome coronavirus 2 (SARS-CoV-2) (1, 2). COVID-19 has proven highly contagious and more lethal due to rapid transmission capabilities and atypical symptoms or even asymptomatic infection (3–5). As of January 8, 2021, 86.4 million confirmed cases of COVID-19 and 1.9 million deaths in total have been reported globally, which still need strong intervention (6).

The pathogenesis and pathophysiology for COVID-19 are still unclear. Immune function may play a vital role in SARS-CoV-2 infection and there is still no specific antiviral drug for the treatment of SARS-CoV-2 until now (7). Much research has shown changes of cytokines, lymphocyte subsets, and the dysregulation of host immune response for patients with different severities of COVID-19 (8–13). However, few studies have involved the complement system, and been conducted based on the clinical courses among patients with definite outcomes (14).

In this retrospective study, we compared clinical, laboratory, and radiological characteristics of cured and deceased patients among all confirmed COVID-19 patients who were transferred or admitted to the designated hospital, Wuhan Tongji Hospital, as of March 30, 2020. We aimed to explore the risk factors for mortality of patients with definite clinical outcomes based on clinical course, especially in the complement system, and inflammatory and immune responses. Finally, we hope to find some clues for new treatment strategies. Our results suggest that decreased C3 and C4, dysregulation of lymphocyte subsets, and cytokines may lead to death after SARS-CoV-2 infection.

## Materials and Methods

### Study Design

**Figure 1:**
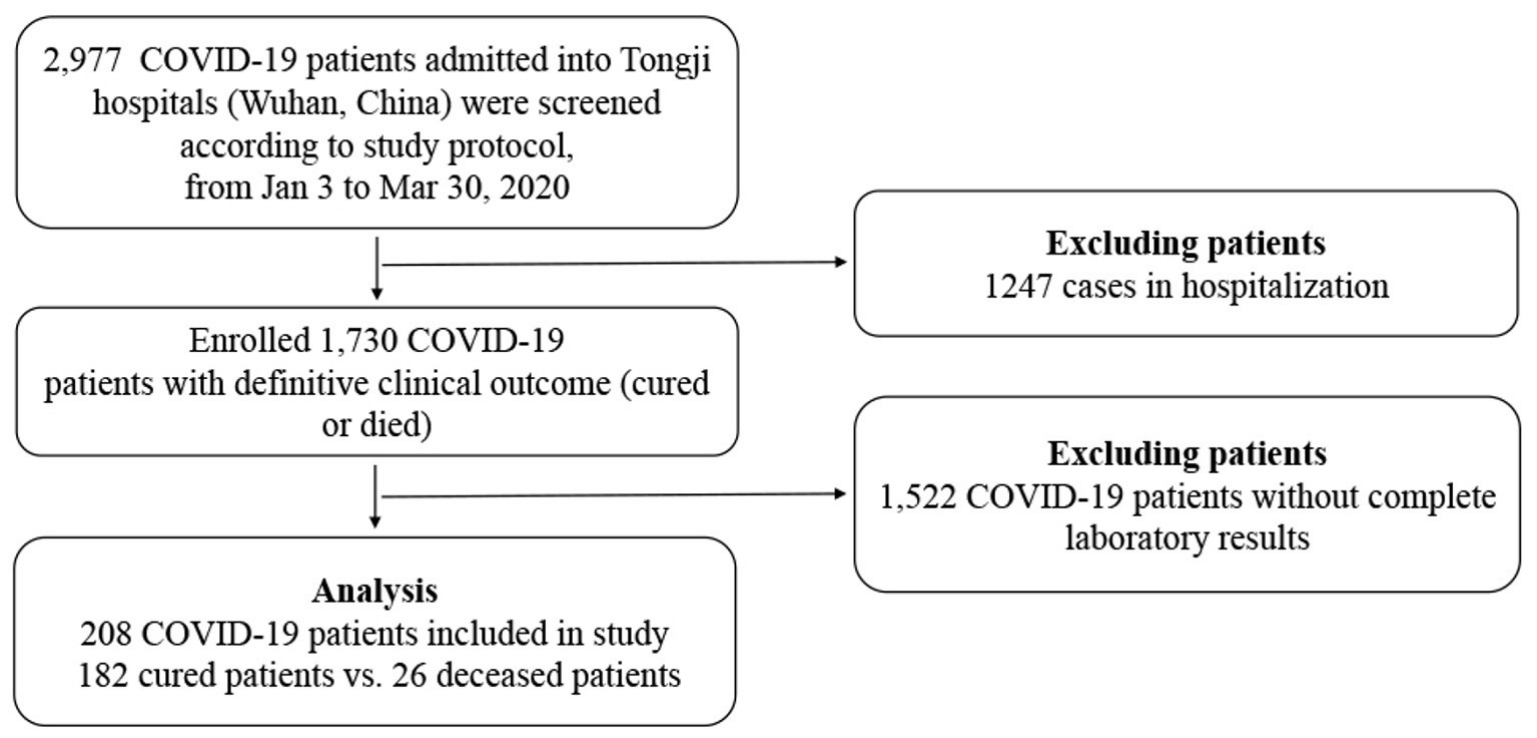
Study flow diagram.

From January 3 to March 30 2020, 2,977 COVID-19 patients were transferred from other designated hospitals, mobile cabin hospitals, isolation sites, or admitted to Tongji Hospital. All adult patients (≥18 years old) were confirmed to have SARS-CoV-2 real-time RT-PCR or serum IgM and IgG antibodies, and diagnosed according to the Seventh Revised Trial Version of the Novel Coronavirus Pneumonia Diagnosis and Treatment Guidance released by the National Health Commission of China (available from http://www.nhc.gov.cn/yzygj/s7653p/202003/46c9294a7dfe4cef80dc7f5912eb1989.shtml). One thousand, seven hundred thirty patients were enrolled with definite clinical outcomes (cured or deceased), but only 208 patients were included with complete laboratory and radiological results (Figure 1). All the cured patients had repeated negative tests for SARS-Cov-2 RNA at least 24 h apart, symptomatic relief, significant improvement in pulmonary radiology, and no need of supportive care or other treatments. Exclusion criteria: (1) missing laboratory or clinical data; (2) Patients still hospitalized; (3) Patients who died in 24 h after hospitalization. The study was approved by the Research Ethics Commission of Tongji Hospital and the requirement for informed consent was waived by the Ethics Commission (IRB ID:TJ- IRB20200343).

### Cell Subsets, Cytokines, and Receptors Testing

In order to quantify T lymphocyte subsets, 1 × 10^6^ PBMCs were stained with indicated antibodies (CD3-APC, CD4-PerCP, and CD8-FITC antibodies all from BD Biosciences) in the dark at room temperature for 20 min. After two washes, the cells were analyzed within 1 h. All samples were detected by BD FACS Canto II Flow Cytometry System and analyzed with the BD FACS Diva Software.

In order to explore the influence of COVID-19 on the secretion of cytokines and receptors, chemiluminescence immunoassay (CLIA) was performed. Cytokines and receptors including interleukin 2 receptor (IL-2R), IL-6, IL-8, IL-10 and TNF-α were assessed using serum samples (Immulite 1000, DiaSorin Liaison, Italy; or Cobas e602, Roche Diagnostics, Germany). The testing kit was purchased from Roche Diagnostics.

### Data Collection

Demographic data, medical history, clinical manifestations, comorbidities, laboratory tests, and radiological findings were extracted from the electronic medical records. The laboratory tests included complete blood count, serum biochemical tests [containing liver and renal function, lactate dehydrogenase (LDH), and electrolytes], myocardial enzymes, NT-proBNP, coagulation profile, procalcitonin (PCT), C-reactive protein (CRP), serum ferritin, erythrocyte sedimentation rate (ESR), cytokines and receptors, lymphocyte subsets, and immune function (including C3 and C4). Chest computed tomography (CT) was done for all patients. The results of virus tests, including SARS-CoV-2 RNA real-time RT-PCR and serum IgM and IgG antibodies, were also recorded.

### Statistical Analysis

We used SPSS (version 23.0) for all statistical analyses. Normally and non-normally distributed continuous variables were expressed as means with standard deviations (SD) or medians with inter-quartile range (IQR), respectively, and analyzed with a Student's *t*-test or Mann–Whitney *U*-test. Categorical variables were presented as number and proportions, and compared with a Chi-square test, or Fisher's test if cells are small in number. Variables with *P* < 0.05 was considered to indicate a statistically significant difference. ROC curves were used to evaluate the prognostic value of the clinical parameters. To explore the independent prognostic factors for mortality, backward stepwise, binary logistic regression analysis was performed. Kaplan–Meier analysis was conducted to further confirm the predictive value of clinical parameters on survival. Spearman correlation analysis was used to evaluate the correlation between the variables. Values of *P* < 0.05 were considered statistically significant.

## Results

### Demographic and Clinical Characteristics of COVID-19 Patients

Among the 208 included patients for the final analysis, 182 patients were cured and discharged, and 26 patients died from COVID-2019. Compared with cured patients, deceased patients were significantly older [median age, 69 (59–78) vs. 64 (52–71) years; *P* = 0.035]. Moreover, the median time of hospitalization in the deceased group was a little bit shorter than cured group [29.0 (17.5–32.5) vs. 32.0 (24.0–41.0) days; *P* = 0.210] although there was no significant difference, and the peak of death occurred 30 to 50 days after onset. Most patients had one or more comorbidities, with hypertension as the most common chronic disease (80; 38%), followed by surgery (39; 19%), diabetes (37; 18%), and allergy (23; 11%). The most common symptoms at onset were fever (149; 72%), cough (137; 66%), and dyspnea (113; 54%), among others including fatigue (37; 18%), diarrhea (36; 17%), sore throat, myalgia, et al., with no significant difference between the two groups. All patients had at least one chest CT test after admission to hospital, with 150 (82%) recovered patients and 24 (92%) deaths had bilateral involvement (*P* = 0.202) (Table 1). Of the 208 included patients, 109/208 (52.4%) patients received corticosteroid therapy.

**Table 1:** Demographic and clinical findings of COVID-19 patients included in the study.

**Indicators**	**Total (*N =* 208)**	**Cured (*N =* 182)**	**Died (*N =* 26)**	***P*-value**
**Characteristics**				
Age, years	64 (53-72)	64 (52-71)	69 (59-78)	0.035
Sex				0.418
Male	129 (62%)	111 (61%)	18 (69%)	
Female	79 (38%)	71 (39%)	8 (31%)	
Hospital stay, days	29.5 (19.0-40.0)	32.0 (24.0-41.0)	29.0 (17.5-32.5)	0.210
**Comorbidities**				
Hypertension	80 (38%)	69 (38%)	11 (42%)	0.667
Diabetes	37 (18%)	33 (18%)	4 (15%)	0.732
CVD	16 (8%)	13 (7%)	3 (12%)	0.431
COPD	6 (3%)	4 (2%)	2 (8%)	0.347
CKD	2 (1%)	1 (1%)	1 (4%)	0.591
Malignancy	10 (5%)	8 (4%)	2 (8%)	0.806
Surgery	39 (19%)	34 (19%)	5 (19%)	0.946
Allergy	23 (11%)	20 (11%)	3 (12%)	0.933
**Initial symptoms**				
Fever	149 (72%)	131 (72%)	18 (69%)	0.771
Cough	137 (66%)	120 (66%)	17 (65%)	0.956
Dyspnea	113 (54%)	97 (53%)	16 (62%)	0.430
Fatigue	37 (18%)	31 (17%)	6 (23%)	0.451
Diarrhea	36 (17%)	30 (16%)	6 (23%)	0.406
Others	50 (24%)	42 (23%)	8 (31%)	0.391
**CT deterioration**				
Bilateral involvement				0.202
Yes	174 (84%)	150 (82%)	24 (92%)	
No	34 (16%)	32 (18%)	2 (8%)	

*CVD, Cardiovascular Disease; COPD, Chronic obstructive pulmonary disease; CKD, Chronic renal disease. Continuous variables were described as median (Interquartile range)*.

### Laboratory Findings of COVID-19 Patients

The laboratory findings on admission in COVID-19 patients were present in Table 2. The deceased cases had higher levels of leukocytes (8.2 vs. 6.4 × 10^9^/L; *P* = 0.025) and neutrophils (7.0 vs. 4.4 × 10^9^/L; *P* = 0.004) counts, lower lymphocytes counts (0.6 vs 1.1 × 10^9^/L; *P* = 0.001), higher neutrophil percentage (83.9 vs. 70.5%; *P* < 0.001) and lower lymphocyte percentage (9.7 vs. 19.0%; *P* < 0.001), lower eosinophils counts (0.0 vs 0.5 × 10^9^/L; *P* < 0.001). Lymphocyte subsets were detected in all patients enrolled, including T cells, B cells, CD4 T cells, CD8 T cells, and NK cells. Compared with cured patients, deceased patients had lower counts and percentage of T cells, CD4 T cells, CD8 T cells, and NK cells, and a higher percentage of B cells (Table 2).

**Table 2:** Laboratory findings of COVID-19 patients.

**Indicators**	**Total (*N =* 208)**	**Cured (*N =* 182)**	**Died (*N =* 26)**	***P*-value**
**Blood cell counts**				
Leucocytes, × 10^8^ per L	6.4 (4.9–8.6)	6.4 (4.8–8.3)	8.2 (5.4–11.6)	0.025
Neutrophils, × 10^8^ per L	4.5 (3.0–6.8)	4.4 (3.0–6.3)	7.0 (3.9–10.1)	0.004
Neutrophil percentage, %	73.0 (60.8–82.6)	70.5 (60.0–80.7)	83.9 (77.2–91.1)	<0.001
Lymphocytes, × 10^8^ per L	1.1 (0.7–1.4)	1.1 (0.7–1.5)	0.6 (0.5–0.9)	0.001
Lymphocyte percentage, %	17.1 (9.9–27.0)	19.0 (11.5–28.5)	9.7 (5.0–14.2)	<0.001
Monocytes, × 10^8^ per L	0.5 (0.4–0.6)	0.5 (0.4–0.7)	0.4 (0.3–0.6)	0.472
Eosinophils, × 10^8^ per L	0.4 (0.0–1.1)	0.5 (0.0–1.1)	0.0 (0.0–0.1)	<0.001
Basophils, × 10^8^ per L	0.1 (0.1–0.3)	0.1 (0.1–0.3)	0.1 (0.0–0.2)	0.250
T cells (CD3+CD19–), /μl	885.0 (505.5–1232.5)	950.0 (667.0–1293.5)	336.0 (191.3–462.5)	<0.001
T cells (CD3+CD19–), %	70.7 (62.3–77.2)	70.7 (63.0–77.3)	70.2 (49.5–77.5)	0.473
B cells (CD3–CD19+), /μl	72.0 (38.5–118.0)	75.0 (42.0–121.0)	45.0 (15.5–81.5)	0.027
B cells (CD3–CD19+), %	12.5 (8.0–18.4)	12.3 (7.8–16.5)	17.9 (8.9–37.1)	0.012
CD4 T cells (CD3+CD4+), /μl	534.5 (330.3–753.3)	580.5 (423.5–794.3)	200.0 (117.0–291.8)	<0.001
CD8 T cells (CD3+CD8+), /μl	292.0 (153.3–414.8)	326.0 (192.5–429.5)	90.0 (58.3–155.0)	<0.001
NK cells (CD3–/CD16+CD56+), /μl	154.5 (82.3–278.0)	187.0 (104.0–293.5)	28.5 (18.3–81.8)	<0.001
T cells+B cells+NK cells, /μl	1245.0 (783.0–1661.0)	1345.0 (963.0–1737.0)	490.0 (310.3–724.8)	<0.001
Hemoglobin, g per L	126.0 (112.3–140.0)	125.5 (114.5–140.3)	133.0 (97.5–140.5)	0.722
**Inflammatory and immunological factors**				
Interleukin 2 receptor, U/mL	731.5 (466.5–1131.0)	725.5 (464.3–1124.3)	919.5 (529.3–16.1.5)	0.157
Interleukin 6, pg/mL	17.6 (4.9–40.9)	12.9 (4.4–37.3)	39.1 (21.6–71.4)	<0.001
Interleukin 8, pg/mL	14.2 (9.3–25.6)	13.4 (9.0–22.3)	23.5 (11.3–41.9)	0.011
Interleukin 10, pg/mL	6.3 (5.0–9.3)	6.4 (5.0–9.3)	6.1 (5.0–18.9)	0.938
TNF-α, pg/mL	10.2 (7.8–13.2)	10.2 (7.7–13.1)	10.0 (7.7–16.8)	0.839
CRP, mg/L	39.1 (8.2–93.5)	33.7 (6.1–85.2)	104.8 (42.1–220.4)	<0.001
ESR, mm/hour	41.0 (20.0–72.0)	42.0 (21.8–71.3)	36.0 (9.5–85.5)	0.992
Ferritin, ng/ml	725.7 (392.9–1395.6)	610.9 (360.0–1244.0)	2022.5 (1075.6–3380.0)	<0.001
C3, g/L	0.9 (0.7–1.0)	0.9 (0.8–1.1)	0.7 (0.5–0.8)	<0.001
C4, g/L	0.2 (0.2–0.3)	0.2 (0.2–0.3)	0.2 (0.1–0.2)	<0.001
IgM, AU/ml	44.6 (12.3–116.5)	44.6 (15.1–100.7)	52.9 (2.6–275.3)	0.986
IgG, AU/ml	167.8 (97.8–211.0)	169.3 (107.2–206.5)	140.7 (21.5–249.7)	0.551
**Coagulation function**				
Prothrombin time, second	14.0 (13.2–14.9)	13.9 (13.1–14.6)	15.5 (14.5–16.8)	<0.001
D-dimer, μg/mL	1.2 (0.6–2.8)	1.1 (0.5–2.5)	2.6 (0.9–7.8)	0.004
Platelets, × 10^8^ per L	199.5 (152.3–274.0)	203.0 (160.8–274.5)	152.0 (102.5–236.5)	0.052
**Organ damage indices**				
Alanine aminotransferase, U/L	24.0 (16.0–43.8)	23.5 (16.0–43.3)	28.0 (15.8–48.0)	0.633
Aspartate aminotransferase, U/L	28.0 (19.3–43.8)	26.0 (19.0–43.0)	36.0 (25.5–51.8)	0.668
Albumin, g/L	33.9 (29.9–38.5)	34.6 (30.2–39.0)	31.7 (28.3–34.5)	0.003
Total bilirubin, μmol/L	9.5 (6.8–14.0)	9.0 (6.5–13.3)	11.8 (9.1–21.1)	0.005
Lactate dehydrogenase, U/L	282.0 (223.5–407.8)	276.5 (216.0–395.0)	357.0 (258.5–512.8)	0.019
CK–MB	0.8 (0.5–1.3)	0.8 (0.4–1.2)	1.8 (0.9–4.9)	<0.001
Hs-cTnI, pg/mL	7.6 (3.3–19.2)	6.8 (3.0–15.8)	44.4 (7.6–106.7)	<0.001
NT-proBNP, pg/mL	294.5 (71.8–824.3)	196.0 (57.3–614.5)	747.5 (387.5–1643.3)	0.109
Blood urea nitrogen, mmol/L	5.3 (3.8–7.6)	4.9 (3.7–6.6)	9.2 (7.9–12.4)	<0.001
Creatinine, μmol/L	77.0 (60.0–94.8)	75.0 (60.0–91.3)	99.5 (62.8–123.3)	0.006
Procalcitonin, ng/mL	0.1 (0.1–0.2)	0.1 (0.1–0.2)	0.2 (0.1–1.0)	<0.001

*T cell, T lymphocyte; B cell, B lymphocyte; CD4 T cell, helper T lymphocyte; CD8 T cell, suppressor T lymphocyte; NK cell, nature killer cell; CRP, C-reactive protein; ESR, Erythrocyte sedimentation rate; C3, complement component 3; C4, complement component 4; IgM, Immunoglobulin M; IgG, Immunoglobulin G; TNF-α, Tumor necrosis factor α; CK-MB, Creatine Kinase Isoenzyme-MB; hs-cTnI, High-sensitivity cardiac troponin I; NT-proBNP, N-terminal pro brain natriuretic peptide. Continuous variables were described as median (Interquartile range)*.

Inflammatory cytokines were much higher in non-survivors, including IL6 (39.1 vs. 12.9 pg/mL; *P* < 0.001), and IL8 (23.5 vs. 13.4 pg/mL; *P* = 0.011). Compared with survivors, deceased patients had higher levels of infection-related biomarkers, such as CRP (104.8 vs. 33.7 ng/mL; *P* < 0.001), and ferritin (2022.5 vs. 610.9 ng/mL; *P* < 0.001). Complements C3 and C4 were significantly lower in non-survivors than survivors [C3 0.7 (0.5–0.8) vs. 0.9 (0.8–1.1), *P* < 0.001; C4 0.2 (0.1–0.2) vs. 0.2 (0.2–0.3), *P* < 0.001] (Table 2). Deceased patients seemed to be prone to dysfunctional coagulation system with longer prothrombin time (PT) (15.5 vs. 13.9 ug/mL; *P* < 0.001), elevated D-dimer (2.6 vs. 1.1 ug/mL; *P* = 0.004), and decreased platelets (152.0 vs. 203.0 ug/mL; *P* = 0.052).

The deceased patients tended to have worse liver function (i.e., higher total bilirubin and lactate dehydrogenase, lower albumin), renal function (increased blood urea nitrogen and creatinine), myocardial enzymes (elevated CK-MB and hs-cTnI), NT-proBNP and severer bacterial secondary lung infections (Procalcitonin) than recovered patients (Table 2).

### Prognostic Value of the Clinical Parameters

Lots of variables were associated with survival by ROC curve analysis as shown in Table 3. Complete blood cell count (leucocyte, neutrophil and lymphocyte count, and percentage), especially lymphocyte subsets (T cells, CD4 T cells, CD8 T cells, NK cells, T cells + B cells + NK cells), inflammatory biomarkers (procalcitonin, CRP, ferritin, IL6, IL8), immunological factors (C3, C4), PT, D-dimer, myocardial enzymes, and blood urea nitrogen (BUN) all exhibited predictive effect for prognosis (Table 3). Among these, C3 (AUC = 0.814; 95% CI: 0.715–0.912; *P* < 0.001) and lymphocyte subsets showed predominant prognostic value.

**Table 3:** Prognostic value of the clinical parameters.

	**AUC**	**95% Confident interval**	***P*-value**
Age	0.628	0.523–0.732	0.035
Albumin	0.679	0.579–0.779	0.003
Total bilirubin	0.669	0.556–0.781	0.005
Lactate dehydrogenase	0.643	0.527–0.759	0.019
CK-MB	0.766	0.659–0.873	<0.001
Hs-cTnI	0.776	0.667–0.886	<0.001
Blood urea nitrogen	0.854	0.779–0.929	<0.001
Creatine	0.667	0.542–0.792	0.006
Prothrombin time	0.798	0.693–0.898	<0.001
D–dimer	0.677	0.574–0.780	0.004
Procalcitonin	0.784	0.696–0.871	<0.001
CRP	0.731	0.615–0.846	<0.001
Ferritin	0.779	0.675–0.882	<0.001
C3	0.814	0.715–0.912	<0.001
C4	0.704	0.578–0.829	0.003
Interleukin 6	0.738	0.648–0.829	<0.001
Interleukin 8	0.655	0.532–0.778	0.011
Leucocytes	0.636	0.507–0.764	0.025
Neutrophils	0.675	0.551–0.799	0.004
Neutrophil percentage	0.753	0.646–0.860	<0.001
Lymphocytes	0.723	0.613–0.833	<0.001
Lymphocyte percentage	0.755	0.656–0.854	<0.001
Eosinophils	0.746	0.662–0.830	<0.001
T cells (CD3+CD19-)	0.909	0.862–0.956	<0.001
B cells (CD3-CD19+)	0.670	0.557–0.784	0.006
B cells (CD3-CD19+) %	0.652	0.519–0.785	0.012
CD4 T cells (CD3+CD4+)	0.899	0.852–0.946	<0.001
CD8 T cells (CD3+CD8+)	0.877	0.814–0.939	<0.001
NK cells (CD3-/CD16+CD56+)	0.900	0.744–0.956	<0.001
T cells+B cells+NK cells	0.915	0.874–0.957	<0.001

*CK-MB, Creatine Kinase Isoenzyme-MB; hs-cTnI, High-sensitivity cardiac troponin I; CRP, C-reactive protein; C3, complement component 3; C4, complement component 4; T cell, T lymphocyte; B cell, B lymphocyte; CD4 T cell, helper T lymphocyte; CD8 T cell, suppressor T lymphocyte; NK cell, nature killer cell*.

**Figure 2:**
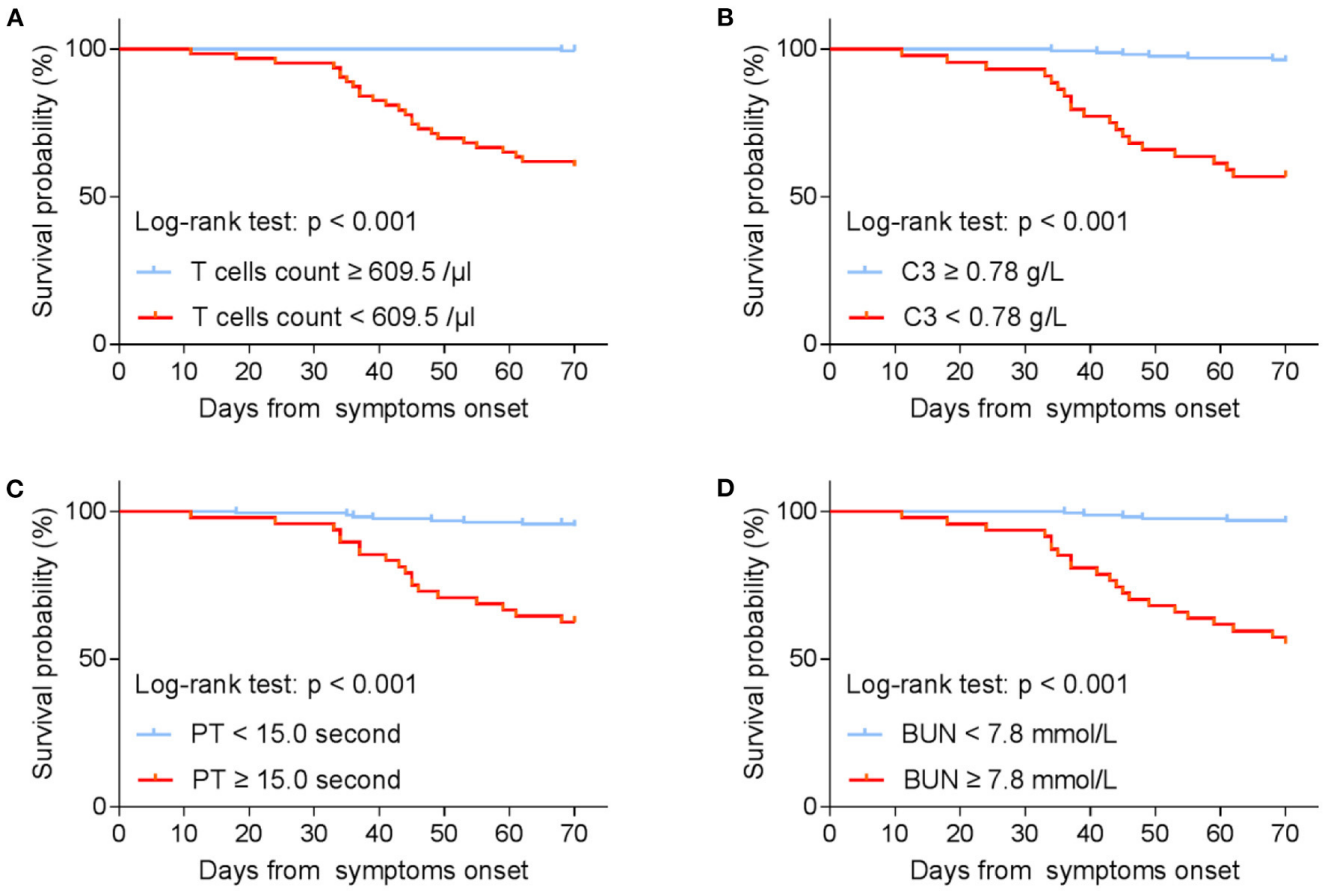
Kaplan–Meier estimates on survival by **(A)** T cells count; **(B)** C3; **(C)** PT; **(D)** BUN (stratified according to best cut-off). T cell, T lymphocyte; C3, complement component 3; PT, Prothrombin time; BUN, Blood urea nitrogen.

In addition, PT (OR = 2.084, 95% CI: 1.157–3.752; *P* = 0.014), T cells (OR = 0.992, 95% CI: 0.987–0.996; *P* < 0.001), and C3 (OR = 0.010, 95% CI: 0.000–0.677; *P* = 0.032) were confirmed as independent prognostic factors for mortality by logistic regression models (Table 4). Kaplan–Meier analysis elucidated that lower levels of T cells and C3, higher PT and BUN were related to poor survival (Figure 2). Hence, T cells and C3 were valuable protective prognostic factors for mortality, whereas PT was revealed as a risk factor.

**Table 4:** Logistic regression of independent prognostic factors for mortality (*r*^2^ = 0.741, *P* <0.001).

**Factors**	**β**	**Sx̄**	***P*-value**	**OR for Mortality (95% CI)**
PT	0.734	0.300	0.014	2.084 (1.157–3.752)
T cells	−0.008	0.002	<0.001	0.992 (0.987–0.996)
C3	−4.641	2.169	0.032	0.010 (0.000–0.677)

*PT, Prothrombin time; T cell, T lymphocyte; C3, complement component 3*.

### Clinical Courses of Two Typical Hospitalized COVID-19 Patients From Illness Onset

**Figure 3:**
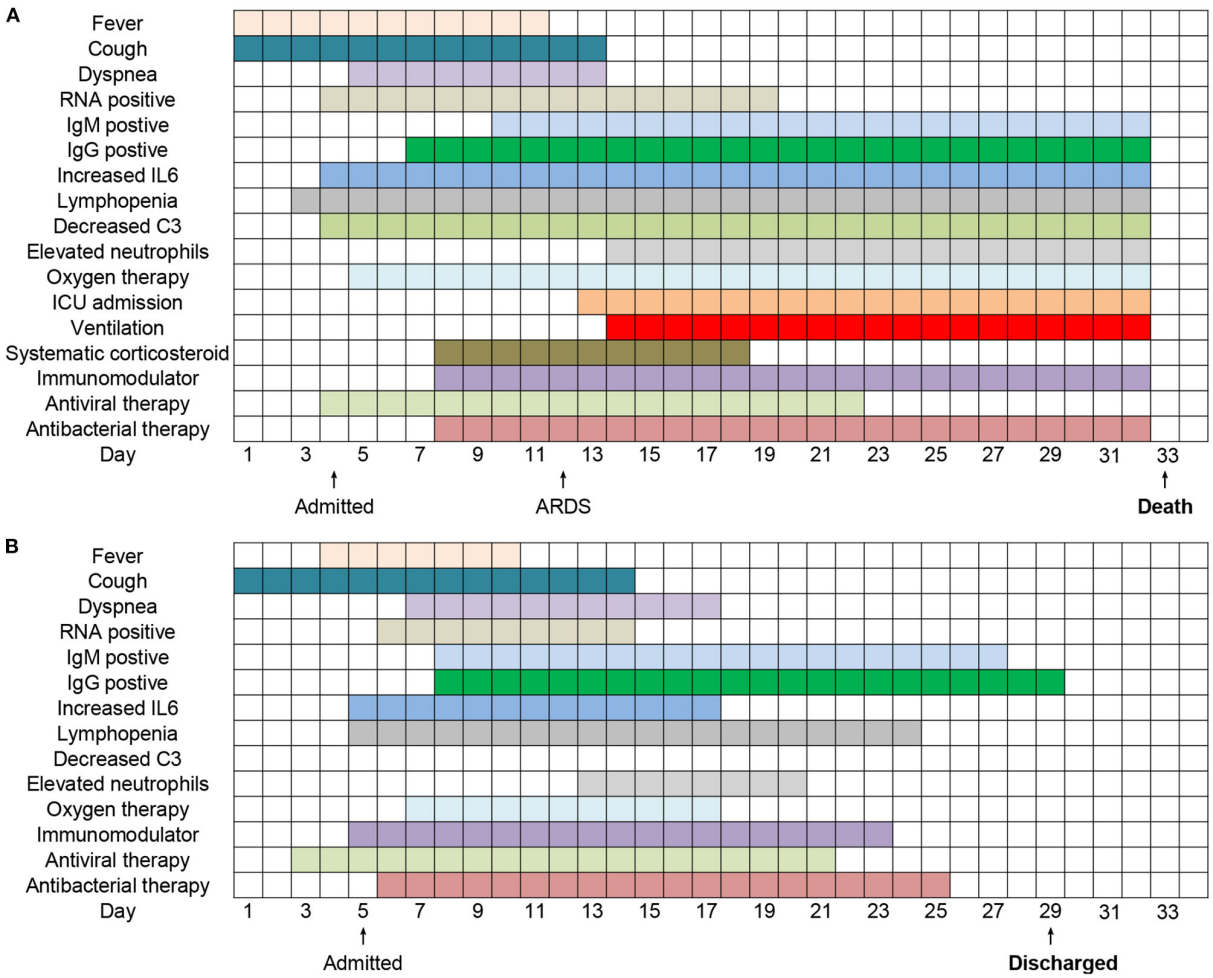
Clinical courses of 2 typical hospitalized patients **(A,B)** with COVID-19 from illness onset.

The duration of clinical symptoms, viral shedding, abnormal laboratory tests, and treatments of two typical hospitalized COVID-19 patients from illness onset were presented in Figure 3. Fever persisted longer in the deceased patients (11 days) than the recovered (7 days). The course of viral shedding in cured cases was much shorter (9 vs. 16 days). Increased IL6, lymphopenia, elevated neutrophils lasted longer in the fatal casea, who also had decreased C3. The initiation time and duration of immunomodulator, antiviral and antibacterial treatments were similar between the two patients. However, the deceased patients received systematic corticosteroid and longer oxygen therapy (25 vs. 11 days), and underwent about 18 days invasive mechanical ventilation and 19 days ICU admission after ARDS.

### Temporal Changes in Laboratory Markers in Hospitalized COVID-19 Patients From Illness Onset

**Figure 4:**
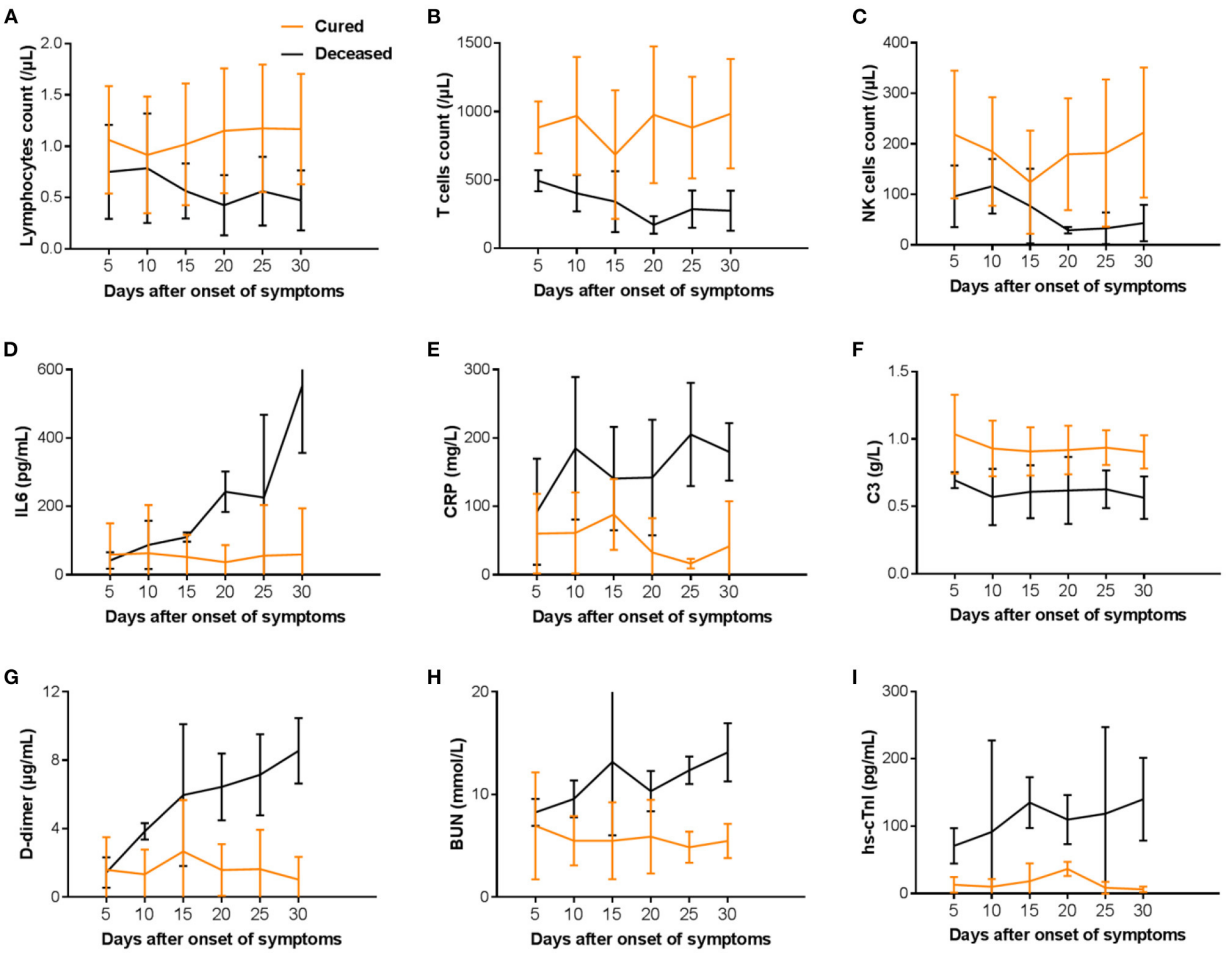
Temporal changes in laboratory markers **(A–I)** in hospitalized patients since onset of COVID-19. T cell, T lymphocyte; B cell, B lymphocyte; NK cell, nature killer cell; IL6, Interleukin 6; CRP, C-reactive protein; C3, complement component 3; BUN, Blood urea nitrogen; hs-cTnI, High-sensitivity cardiac troponin I.

The trend of key laboratory markers was tracked after illness onset (Figure 4). In the non-survivors, lymphocytes, T cells, and NK cells counts were significantly lower than survivors, with the worst observed around day 20 until death. In survivors, the severe lymphopenia was occurred on day 10, T cells and NK cells count were lowest on day 15, and could gradually return to normal during hospitalization (Figures 4A–C). Levels of IL6, CRP, D-dimer, BUN, and hs-cTnI were significantly elevated in deaths compared with recovered patients throughout the clinical course, whereas C3 decreased with disease exacerbation (Figures 4D–I). In the deceased patients, IL6 increased rapidly on day 25 from disease onset (Figure 4D), while D-dimer, BUN, and hs-cTnI increased rapidly in the early stage of illness (Figures 4G–I). Overall, the levels of inflammatory factors between the two groups showed apparent difference even in the first 20 days from illness onset.

### Correlation Analysis Between Blood Cell Counts, Organ Damage Indices and Inflammatory Factors

Correlation analysis between blood cell counts, organ damage indices and inflammatory factors (IL6, IL8, C3, C4, CRP, and ferritin) were evaluated by the Spearman test (Table 5). IL6 and CPR were positively correlated with neutrophils (*r* = 0.242, *P* < 0.01; *r* = 0.414, *P* < 0.01), but negatively with lymphocytes (*r* = −0.255, *P* < 0.01; *r* = −0.380, *P* < 0.01) and lymphocyte subsets except B cells. Meanwhile, IL8 and ferritin were negatively related to T cells (*r* = −0.162, *P* < 0.05; *r* = −0.197, *P* < 0.05) and CD4 T cells (*r* = −0.161, *P* < 0.05; *r* = −0.207, *P* < 0.05). Positive associations existed between C3 and T cells (*r* = 0.374, *P* < 0.01), CD4 T cells (*r* = 0.367, *P* < 0.01) and CD8 T cells (*r* = 0.331, *P* < 0.01), whereas no significant correlation was found between C4 and lymphocyte subsets (Table 5). In addition, PT was found to be positively correlated with IL6 (*r* = 0.155, *P* < 0.05), IL8 (*r* = 0.191, *P* < 0.01) and CRP (*r* = 0.318, *P* < 0.01). Reverse correlations were explored between C3, C4 and PT, CK-MB, total bilirubin, as well as C3 and BUN, procalcitonin. Moreover, CRP was positively correlated with D-dimer (*r* = 0.238, *P* < 0.01), hs-cTnI (*r* = 0.209, *P* < 0.01), and BUN (*r* = 0.225, *P* < 0.01), as well as ferritin and BUN.

**Table 5:** Correlation analysis was evaluated between blood cell counts, organ damage indices, and inflammatory factors.

**Indicators**	**Inflammatory factors**					
	**IL6**	**IL8**	**C3**	**C4**	**CRP**	**Ferritin**
**Blood cell counts**					
Neutrophils	0.224^**^	0.111	−0.020	−0.036	0.354^**^	0.259^**^
Monocyte	−0.018	−0.067	0.093	−0.016	−0.035	0.010
Eosinophils	−0.282^**^	−0.074	0.227^*^	0.010	−0.358^**^	−0.272^**^
Lymphocytes	−0.402^**^	−0.259^**^	0.201^*^	−0.025	−0.447^**^	−0.313^**^
T cells	−0.369^**^	−0.218^*^	0.418^**^	0.104	−0.288^**^	−0.311^**^
B cells	−0.253^**^	−0.182^**^	0.130	−0.014	−0.193^**^	−0.103
CD4 T cells	−0.384^**^	−0.225^*^	0.411^**^	0.140	−0.312^**^	−0.296^**^
CD8 T cells	−0.288^**^	−0.176^*^	0.375^**^	0.056	−0.212^**^	−0.313^**^
NK cells	−0.291^*^	−0.108	0.216^*^	0.086	−0.297^**^	−0.278^**^
**Organ damage indices**					
PT	0.325^**^	0.250^**^	−0.300^**^	−0.196^*^	0.476^**^	0.339^**^
D-dimer	0.283^**^	0.257^**^	−0.003	−0.109	0.445^**^	0.311^**^
CK-MB	0.176^*^	0.269^**^	−0.376^**^ss	−0.241^*^	0.060	0.023
Hs-cTnI	0.282^**^	0.248^**^	−0.286^**^	−0.154	0.282^**^	0.160
Total bilirubin	0.335^**^	0.264^**^	−0.198^*^	−0.250^**^	0.345^**^	0.347^**^
BUN	0.233^**^	0.259^**^	−0.248^**^	0.013	0.292^**^	0.301^**^
Procalcitonin	0.459^**^	0.326^**^	−0.252^**^	−0.120	0.585^**^	0.525^**^

*Data expressed as correlation coefficient*.

* *P < 0.05*;

** *P < 0.01*.

*T cell, T lymphocyte; B cell, B lymphocyte; CD4 T cell, helper T lymphocyte; CD8 T cell, suppressor T lymphocyte; NK cell, nature killer cell; PT, Prothrombin time; CK-MB, Creatine Kinase Isoenzyme-MB; hs-cTnI, High-sensitivity cardiac troponin I; BUN, Blood urea nitrogen; IL6, Interleukin 6; IL8, Interleukin 8; C3, complement component 3; CRP, C-reactive protein*.

## Discussion

Although the changes in the hematological tests, infection-related markers, and lymphocyte subsets of COVID-19 patients were described by lots of literature (9–13), few studies have investigated the trend through the clinical process with definite outcomes (14). In this retrospective case-control study, we reported inflammatory and immunological factors and lymphocyte subsets in the cohort of 208 COVID-19 patients with definite outcomes. Collectively, increased inflammatory cytokines, T lymphopenia, especially CD4 T cells (CD3+CD4+, abbreviated as CD4+) and CD8 T cells (CD3+CD8+, abbreviated as CD8+), decreased C3 and C4, were more pronounced in the deceased patients. Decreased C3 and C4, dysregulation of lymphocyte subsets and cytokines, may lead to death after SARS-CoV-2 infection.

We noticed that complement C3 and C4 were reduced in the non-survivors, indicating that the dysregulated complement system may be activated in COVID-19. To our knowledge, the complement system is critical in innate immune response to viral infections (15–17). Several studies have investigated the role of the complement system in SARS-CoV infection and demonstrated that the activation of the complement system can induce illness deterioration. Gralinski revealed that SARS-CoV infected C3-deficient mice had lower levels of neutrophils, monocytes, immune cells, and cytokines than SARS-CoV infected controls, and concluded that in the absence of complement, SARS-CoV was unable to lead to inflammatory response as in wild-type mice (17). Risitano indicated that decreased C3 could contribute to thrombus, inflammation, and organ damage in COVID-19 (18). Recent clinical findings showed the highly selective C3-target inhibitor, AMY-101, with good safety and tolerability in a Phase I study, and the Phase II clinical trials are currently underway (19, 20). Therefore, C3 inhibition may be a prospective therapeutic approach to inhibit complement activation, and complement-mediated inflammatory and immune response in COVID-19 patients.

The significant elevation of IL6, a pro-inflammatory cytokine, can lead to a “cytokine storm” and result in acute lung injury, acute respiratory distress syndrome (ARDS), MODS or even multiple organ failure (MOF) (10, 12, 21). Coronavirus infection of macrophages, dendritic cells, and monocytes induce their own activation and promote the secretion of IL6 and other cytokines. IL6 can function through both classic cis signaling and trans signaling pathways (10, 22). In addition, IL6 could bind to membrane-bound IL6 receptor (mIL-6R)–gp130 complex in cis signaling, and soluble form of IL6R (sIL-6R) with a gp130 dimer in trans signaling, and then activate downstream signal transduction through JAKs (Janus kinases) and STAT3 (signal transducer and activator of transcription three). Activation of cis signaling can lead to multiple effects on the innate and acquired immune system and result in cytokine release syndrome (CRS) as mIL-6R is mainly expressed on the surface of immune cells (10, 22). Trans signaling is always triggered in cells that do not express mIL-6R, like endothelial cells, leading to secretion of additional IL6, IL8, vascular endothelial growth factor (VEGF), and monocyte chemoattractant protein-1 (MCP-1), reduction of E-cadherin, and finally giving rise to a “cytokine storm.” Moreover, IL6 can induce the expression of C-reactive protein (CRP), which is an important inflammatory factor. All of the above further confirm the vital role of IL6 in inflammation and immune response. IL-6 may play a key role in driving the COVID-19 hyper-inflammation, so blockade of IL-6 signaling is promising to improve survival efficiency.

Reduced CD4 + and CD8 + T cells were found in SARS infected patients compared with healthy individuals (23, 24). Consistent with previous research (25), we also found that CD4 + and CD8 + T cells were significantly decreased in non-surviving COVID-19 patients in comparison to survivors. The critical role of CD8+ T cells has been proven in respiratory viral clearance (26, 27), and CD8+ T cell response was related to the severity of Middle East respiratory syndrome coronavirus (MERS-CoV) infection (28). Effective T cell responses can help effectively eliminate MERS-CoV and control high levels of inflammatory cytokines that can cause extensive inflammatory response and even pulmonary injury. In the early stage of infection, CD4+ regulatory T cells are essential for activating CD8+ T cells to respond to acute respiratory virus infection (29). Elevated inflammatory cytokines, and loss of CD4+ and CD8+ T cells in the deceased COVID-19 patients, may cause strong inflammation, cytokine storms, and eventually more severe tissue damage. Therefore, dysregulated immune responses with consumption of lymphocytes may play a pivotal role for COVID-19 pathogenesis.

Previous studies showed that higher D-dimer and prolonged PT always existed in more severe COVID-19 patients (30, 31). In our study, we also found that D-dimer and PT were higher in deceased patients as compared to the cured. Increased D-dimer, prolonged PT, and thrombocytopenia were recently reported to be associated with 28-day mortality in patients with severe COVID-19 infection (32), and the combination of these could be explained by the coagulation system activation and always indicated the possibility of disseminated intravascular coagulation (DIC) (33). Viral infections can lead to inflammatory response and imbalance between procoagulant and anticoagulant homeostasis (34). The inflammatory response induced cytokine storm elicits vascular endothelial dysfunction, disrupts the balance between the coagulation and fibrinolytic systems, and as a result leads to DIC and microcirculation disorder and finally multiple organ dysfunction syndrome (MODS) (35, 36). Besides endothelial damage, additional pathogenetic mechanisms, including increased von Willebrand factor, and activated Toll-like receptor and tissue-factor pathways, are also involved (37). Hence, early detection and routine monitoring of hemostasis tests, and correction of coagulation dysfunction in time, could help to effectively establish treatment strategy and reduce mortality (38, 39). D-dimer is the degradation product of fibrin, with the elevated level indicating hypercoagulating state and secondary fibrinolysis, while PT is related to exogenous coagulating system. Both can be used for early diagnosis of coagulopathy. Our study reinforced that coagulation dysfunction was more likely to occur in decreased COVID-19 patients, and PT could be used as an independent indicator to predict the mortality for COVID-19.

There were several limitations in this study. First, this was a retrospective, single center study of patients admitted to Wuhan Tongji Hospital. Multicenter research may be needed to evaluate the temporal changes of laboratory markers with COVID-19. Second, some patients might lack laboratory tests in the defined time intervals due to the retrospective study design, which would affect the estimation of their role in predicting mortality. Third, the COVID-19 patients with bacterial co-infection or sepsis might affect the outcome of immune response. Last but not least, our findings may be limited by sample size. The results should be validated in a prospective study with a larger sample.

## Conclusions

In summary, our study demonstrated that T cells, C3, and PT were independent prognostic factors for the mortality of patients with COVID-19. The dysregulated inflammation and immune response in COVID-19 patients may be mainly due to lymphopenia, especially CD4 + and CD8 + T lymphopenia, activation of complement system, extensive inflammation, production of cytokine storm, and eventually severe multi-organ injury. Taken together, surveillance of lymphocyte subsets, inflammatory, and immunological factors would effectively help clinicians to predict the prognosis, and make proper strategies for patients with COVID-19.

## Data Availability Statement

The raw data supporting the conclusions of this article will be made available by the authors, without undue reservation.

## Ethics Statement

The studies involving human participants were reviewed and approved by Research Ethics Commission of Tongji Hospital. Written informed consent for participation was not required for this study in accordance with the national legislation and the institutional requirements.

## Author Contributions

CY made substantial contributions to the study design and took responsibility for obtaining ethical approval. JZ was in charge of the manuscript draft. CY and XW took responsibility for data acquisition. JZ and ZW made main contributions to data analysis and interpretation. CY, JZ, and XW participated in the diagnosis and treatment of health professionals. ZW, ZH, and PL made substantial revisions to the manuscript. All authors contributed to the article and approved the submitted version.

## Conflict of Interest

The authors declare that the research was conducted in the absence of any commercial or financial relationships that could be construed as a potential conflict of interest.

## References

[1] Zhu N Zhang D Wang W Li X Yang B Song J. A novel coronavirus from patients with pneumonia in China, 2019. N Engl J Med. (2020) 382:727–33. 10.1056/NEJMoa2001017 31978945 PMC7092803

[2] Chan JF Kok KH Zhu Z Chu H To KK Yuan S. Genomic characterization of the 2019 novel human-pathogenic coronavirus isolated from a patient with atypical pneumonia after visiting Wuhan. Emerg Microbes Infect. (2020) 9:221–36. 10.1080/22221751.2020.1719902 31987001 PMC7067204

[3] Li Q Guan X Wu P Wang X Zhou L Tong Y. Early transmission dynamics in Wuhan, China, of novel coronavirus-infected pneumonia. N Engl J Med. (2020) 382:1199–207. 10.1056/NEJMoa2001316 31995857 PMC7121484

[4] Rothe C Schunk M Sothmann P Bretzel G Froeschl G Wallrauch C. Transmission of 2019-nCoV infection from an asymptomatic contact in Germany. N Engl J Med. (2020) 382:970–1. 10.1056/NEJMc2001468 32003551 PMC7120970

[5] Chan JF Yuan S Kok KH To KK Chu H Yang J. A familial cluster of pneumonia associated with the 2019 novel coronavirus indicating person-to-person transmission: a study of a family cluster. Lancet. (2020) 395:514–23. 10.1016/S0140-6736(20)30154-9 31986261 PMC7159286

[6] W. H. Organization. WHO Coronavirus Disease (COVID-19) Dashboard. Available online at: https://covid19.who.int/

[7] Lin L Lu L Cao W Li T. Hypothesis for potential pathogenesis of SARS-CoV-2 infection-a review of immune changes in patients with viral pneumonia. Emerg Microbes Infect. (2020) 9:727–32. 10.1080/22221751.2020.1746199 32196410 PMC7170333

[8] Huang C Wang Y Li X Ren L Zhao J Hu Y. Clinical features of patients infected with 2019 novel coronavirus in Wuhan, China. Lancet. (2020) 395:497–506. 10.1016/S0140-6736(20)30183-5 31986264 PMC7159299

[9] Wang D Hu B Hu C Zhu F Liu X Zhang J. Clinical characteristics of 138 hospitalized patients with 2019 novel coronavirus-infected pneumonia in Wuhan, China. JAMA. (2020) 323:1061–9. 10.1001/jama.2020.1585 32031570 PMC7042881

[10] Moore JB June CH. Cytokine release syndrome in severe COVID-19. Science. (2020) 368:473–4. 10.1126/science.abb8925 32303591

[11] Qin C Zhou L Hu Z Zhang S Yang S Tao Y. Dysregulation of immune response in patients with COVID-19 in Wuhan, China. Clin Infect Dis. (2020) 71:762–8. 10.1093/cid/ciaa248 32161940 PMC7108125

[12] Chen G Wu D Guo W Cao Y Huang D Wang H. Clinical and immunological features of severe and moderate coronavirus disease 2019. J Clin Invest. (2020) 130:2620–9. 10.1172/JCI137244 32217835 PMC7190990

[13] Tian J Yuan X Xiao J Zhong Q Yang C Liu B. Clinical characteristics and risk factors associated with COVID-19 disease severity in patients with cancer in Wuhan, China: a multicentre, retrospective, cohort study. Lancet Oncol. (2020) 21:893–903. 10.1016/S1470-2045(20)30309-0 32479790 PMC7259911

[14] Zhou F Yu T Du R Fan G Liu Y Liu Z. Clinical course and risk factors for mortality of adult inpatients with COVID-19 in Wuhan, China: a retrospective cohort study. Lancet. (2020) 395:1054–62. 10.1016/S0140-6736(20)30566-3 32171076 PMC7270627

[15] Maglakelidze N Manto KM Craig TJ. a review: does complement or the contact system have a role in protection or pathogenesis of COVID-19? Pulm Ther. (2020) 6:169–76. 10.1007/s41030-020-00118-5 32405877 PMC7218701

[16] Li G Fan Y Lai Y Han T Li Z Zhou P. Coronavirus infections and immune responses. J Med Virol. (2020) 92:424–32. 10.1002/jmv.25685 31981224 PMC7166547

[17] Gralinski LE Sheahan TP Morrison TE Menachery VD Jensen K Leist SR. Complement activation contributes to severe acute respiratory syndrome coronavirus pathogenesis. mBio. (2018) 9:e01753-18. 10.1128/mBio.01753-18 30301856 PMC6178621

[18] Mastaglio S Ruggeri A Risitano AM Angelillo P Yancopoulou D Mastellos DC. The first case of COVID-19 treated with the complement C3 inhibitor AMY-101. Clin Immunol. (2020) 215:108450. 10.1016/j.clim.2020.108450 32360516 PMC7189192

[19] Hajishengallis G Kajikawa T Hajishengallis E Maekawa T Reis ES Mastellos DC. Complement-dependent mechanisms and interventions in periodontal disease. Front Immunol. (2019) 10:406. 10.3389/fimmu.2019.00406 30915073 PMC6422998

[20] Reis ES Berger N Wang X Koutsogiannaki S Doot RK Gumas JT. Safety profile after prolonged C3 inhibition. Clin Immunol. (2018) 197:96–106. 10.1016/j.clim.2018.09.004 30217791 PMC6258316

[21] Ruan Q Yang K Wang W Jiang L Song J. Clinical predictors of mortality due to COVID-19 based on an analysis of data of 150 patients from Wuhan, China. Intensive Care Med. (2020) 46:846–8. 10.1007/s00134-020-05991-x 32125452 PMC7080116

[22] Kang S Tanaka T Narazaki M Kishimoto T. Targeting interleukin-6 signaling in clinic. Immunity. (2019) 50:1007–23. 10.1016/j.immuni.2019.03.026 30995492

[23] Li T Qiu Z Zhang L Han Y He W Liu Z. Significant changes of peripheral T lymphocyte subsets in patients with severe acute respiratory syndrome. J Infect Dis. (2004) 189:648–51. 10.1086/381535 14767818 PMC7109946

[24] Wong RS Wu A To KF Lee N Lam CW Wong CK. Haematological manifestations in patients with severe acute respiratory syndrome: retrospective analysis. BMJ. (2003) 326:1358–62. 10.1136/bmj.326.7403.1358 12816821 PMC162124

[25] Xu Z Shi L Wang Y Zhang J Huang L Zhang C. Pathological findings of COVID-19 associated with acute respiratory distress syndrome. Lancet Respir Med. (2020) 8:420–2. 10.1016/S2213-2600(20)30076-X 32085846 PMC7164771

[26] Baazim H Schweiger M Moschinger M Xu H Scherer T Popa A. CD8(+) T cells induce cachexia during chronic viral infection. Nat Immunol. (2019) 20:701–10. 10.1038/s41590-019-0397-y 31110314 PMC6531346

[27] Schmidt ME Varga SM. The CD8 T cell response to respiratory virus infections. Front Immunol. (2018) 9:678. 10.3389/fimmu.2018.00678 29686673 PMC5900024

[28] Shin HS Kim Y Kim G Lee JY Jeong I Joh JS. Immune responses to middle east respiratory syndrome coronavirus during theacute and convalescent phases of human infection. Clin Infect Dis. (2019) 68:984–92. 10.1093/cid/ciy595 30060038 PMC7108191

[29] Rogers MC Lamens KD Shafagati N Johnson M Oury TD Joyce S. CD4(+) Regulatory T cells exert differential functions during early and late stages of the immune response to respiratory viruses. J Immunol. (2018) 201:1253–66. 10.4049/jimmunol.1800096 29997123 PMC6089537

[30] Wu C Chen X Cai Y Xia J Zhou X Xu S. Risk factors associated with acute respiratory distress syndrome and death in patients with coronavirus disease 2019 pneumonia in Wuhan, China. JAMA Intern Med. (2020) 180:934–43. 10.1001/jamainternmed.2020.0994 32167524 PMC7070509

[31] Chen T Wu D Chen H Yan W Yang D Chen G. Clinical characteristics of 113 deceased patients with coronavirus disease 2019: retrospective study. BMJ. (2020) 368:m1091. 10.1136/bmj.m1091 32217556 PMC7190011

[32] Tang N Bai H Chen X Gong J Li D Sun. Anticoagulant treatment is associated with decreased mortality in severe coronavirus disease 2019 patients with coagulopathy. J Thromb Haemost. (2020) 18:1094–9. 10.1111/jth.14817 32220112 PMC9906401

[33] Levi M Thachil J Iba T Levy JH. Coagulation abnormalities and thrombosis in patients with COVID-19. Lancet Haematol. (2020) 7:e438–40. 10.1016/S2352-3026(20)30145-9 32407672 PMC7213964

[34] Giannis D Ziogas IA Gianni P. Coagulation disorders in coronavirus infected patients: COVID-19, SARS-CoV-1, MERS-CoV and lessons from the past. J Clin Virol. (2020) 127:104362. 10.1016/j.jcv.2020.104362 32305883 PMC7195278

[35] Cohen J. The immunopathogenesis of sepsis. Nature. (2002) 420:885–91. 10.1038/nature01326 12490963

[36] Long H Nie L Xiang X Li H Zhang X Fu X. D-Dimer and prothrombin time are the significant indicators of severe COVID-19 and poor prognosis. Biomed Res Int. (2020) 2020:6159720. 10.1155/2020/6159720 32596339 PMC7301188

[37] Key NS Vercellotti GM Winkelmann JC Moldow CF Goodman JL Esmon NL. Infection of vascular endothelial cells with herpes simplex virus enhances tissue factor activity reduces thrombomodulin expression. Proc Natl Acad Sci USA. (1990) 87:7095–9. 10.1073/pnas.87.18.7095 2169619 PMC54690

[38] Han H Yang L Liu R Liu F Wu KL Li J. Prominent changes in blood coagulation of patients with SARS-CoV-2 infection. Clin Chem Lab Med. (2020) 58:1116–20. 10.1515/cclm-2020-0188 32172226

[39] Lippi G Plebani M. Laboratory abnormalities in patients with COVID-2019 infection. Clin Chem Lab Med. (2020) 58:1131–4. 10.1515/cclm-2020-0198 32119647

